# The composition of commercially available human embryo culture media

**DOI:** 10.1093/humrep/deae248

**Published:** 2024-11-25

**Authors:** M S Zagers, M Laverde, M Goddijn, J J de Groot, F A P Schrauwen, F M Vaz, S Mastenbroek

**Affiliations:** Center for Reproductive Medicine, Amsterdam UMC Location University of Amsterdam, Amsterdam, The Netherlands; Amsterdam Reproduction and Development Research Institute, Amsterdam, The Netherlands; Center for Reproductive Medicine, Amsterdam UMC Location University of Amsterdam, Amsterdam, The Netherlands; Amsterdam Reproduction and Development Research Institute, Amsterdam, The Netherlands; Center for Reproductive Medicine, Amsterdam UMC Location University of Amsterdam, Amsterdam, The Netherlands; Laboratory General Clinical Chemistry, Department of Clinical Chemistry, Amsterdam UMC Location University of Amsterdam, Amsterdam, The Netherlands; Laboratory General Clinical Chemistry, Department of Clinical Chemistry, Amsterdam UMC Location University of Amsterdam, Amsterdam, The Netherlands; Laboratory Genetic Metabolic Diseases, Department of Laboratory Medicine and Pediatrics, Emma Children’s Hospital, Amsterdam UMC Location University of Amsterdam, Amsterdam, The Netherlands; Amsterdam Gastroenterology Endocrinology Metabolism, Inborn Errors of Metabolism, Amsterdam, The Netherlands; Core Facility Metabolomics, Amsterdam UMC Location University of Amsterdam, Amsterdam, The Netherlands; Center for Reproductive Medicine, Amsterdam UMC Location University of Amsterdam, Amsterdam, The Netherlands; Amsterdam Reproduction and Development Research Institute, Amsterdam, The Netherlands

**Keywords:** embryo culture medium, composition, culture environment, human preimplantation embryo, IVF/ICSI

## Abstract

**STUDY QUESTION:**

What is the composition of currently available commercial human embryo culture media provided by seven suppliers, for each stage of human preimplantation embryo development?

**SUMMARY ANSWER:**

While common trends existed across brands, distinct differences in composition underlined the absence of a clear standard for human embryo culture medium formulation.

**WHAT IS KNOWN ALREADY:**

The reluctance of manufacturers to fully disclose the composition of their human embryo culture media generates uncertainty regarding the culture conditions that are used for human preimplantation embryo culture. The critical role of the embryo culture environment is well-recognized, with proven effects on IVF success rates and child outcomes, such as birth weight. The lack of comprehensive composition details restricts research efforts crucial for enhancing our understanding of its impacts on these outcomes. The ongoing demand for greater transparency remains unmet, highlighting a significant barrier in embryo culture medium optimization.

**STUDY DESIGN, SIZE, DURATION:**

For this study, 47 different human embryo culture media and protein supplements were purchased between December 2019 and June 2020; they comprise complete media (n = 23), unsupplemented media (n = 14), and supplements (n = 10). Unsupplemented media were supplemented with each available supplement from the same brand (n = 33 combinations). All samples were directly frozen in liquid nitrogen and stored at −80°C until composition analysis.

**PARTICIPANTS/MATERIALS, SETTING, METHODS:**

We determined the concentrations of 40 components in all samples collected (n = 80). Seven electrolytes (calcium, chloride, iron, magnesium, phosphate, potassium, sodium), glucose, immunoglobulins A, G, and M (IgA, IgG, IgM), uric acid, alanine aminotransferase (ALAT), aspartate aminotransferase (ASAT), and albumin, as well as the total protein concentration, were determined in each sample using a Cobas 8000 Analyser (Roche Diagnostics). Analysis of pyruvate, lactate, carnitine, and 21 amino acids was achieved with Ultra-High Performance Liquid Chromatography-Mass Spectrometry (UPLC-MS/MS).

**MAIN RESULTS AND THE ROLE OF CHANCE:**

Our analysis showed that generally, the concentrations of components of ready-to-use human embryo culture media align with established assumptions about the changing needs of an embryo during early development. For instance, glucose concentrations displayed a high-low-high pattern in sequential media systems from all brands: 2.5–3 mM in most fertilization media, 0.5 mM or below in all cleavage stage media, and 2.5–3.3 mM in most blastocyst stage media. Continuous media generally resembled glucose concentrations of cleavage stage media. However, for other components, such as lactate, glycine, and potassium, we observed clear differences in medium composition across different brands. No two embryo culture media compositions were the same. Remarkably, even embryo culture media from brands that belong to the same parent company differed in composition. Additionally, the scientific backing for the specific concentrations used and the differences in the composition of sequential media is quite limited and often based on minimal *in vivo* studies of limited sample size or studies using animal models.

**LARGE SCALE DATA:**

N/A.

**LIMITATIONS, REASONS FOR CAUTION:**

We used a targeted approach and performed a selection of tests which limit the composition analysis to this set of analytes.

**WIDER IMPLICATIONS OF THE FINDINGS:**

Comprehensive disclosure and complete transparency concerning the composition of human embryo culture media, including the exact concentration of each component, are crucial for evidence-based improvements of culture media for human preimplantation embryos.

**STUDY FUNDING/COMPETING INTEREST(S):**

This research was supported by ZonMw (https://www.zonmw.nl/en), Programme Translational Research 2 (project number 446002003). M.G. declares an unrestricted research grant from Ferring not related to the presented work, paid to the institution VU Medical Center. The remaining authors have no conflicts of interest to declare.

**TRIAL REGISTRATION NUMBER:**

N/A.

## Introduction

In the early days of human IVF, preimplantation embryos were cultured in fairly simple salt solutions prepared by embryologists themselves. Nowadays, it is common to purchase more complex commercial embryo culture media from various suppliers (Chronopoulou and Harper, 2015). Although a lot remains unknown, improved understanding of the environmental and nutritional needs of embryos during the preimplantation phase has led to changes in the composition of embryo culture media to better meet those needs (Summers and Biggers, 2003; Chronopoulou and Harper, 2015). Two distinct approaches have been used to determine the needs of human preimplantation embryos and subsequently the human embryo culture media composition. One approach follows the ‘back to nature’ principle that embryo culture media should closely resemble the changing natural *in vivo* embryo environment (Leese, 1998). Variable concentrations of components determined in human oviduct and uterine fluid led to the development of a sequential embryo culture system (Gardner *et al.*, 1996; Gardner, 1998), where each sequential medium provides a different nutritional environment for each stage of preimplantation embryo development. The second approach follows the ‘let the embryo choose’ principle that preimplantation embryos utilize the nutrients they need from an environment where all required nutrients for each stage are available within a tolerated range (Biggers, 1998). This concept supports the use of a single medium to provide an *in vitro* environment in which embryos can develop until the blastocyst stage without medium refreshment, to reduce stress. Both sequential and continuous medium systems are widely used in IVF laboratories and multiple suppliers have both types of embryo culture media available.

Clinical trials comparing human embryo culture media have demonstrated that the choice of culture medium affects IVF efficacy (Mantikou *et al.*, 2013; Youssef *et al.*, 2015; Kleijkers *et al.*, 2016), as well as the foetal growth and birth weight of children conceived through IVF (Dumoulin *et al.*, 2010; Nelissen *et al.*, 2012; Kleijkers *et al.*, 2016). The subsequent challenge of selecting an embryo culture medium is further complicated by uncertainties regarding the media compositions and any modifications to these compositions over time, due to the lack of transparency by manufacturers. Although an ingredient list is frequently provided, these lists are not always complete (Dyrlund *et al.*, 2014; Tarahomi *et al.*, 2019). Moreover, despite multiple calls for transparency, manufacturers refrain from disclosing a complete description of the composition of the embryo culture media, including the concentrations of all components, for commercial reasons (Biggers, 2000; Evers, 2016; Sunde *et al.*, 2016; Tarahomi *et al.*, 2019; Paulson, 2023). As a result, the exact culture conditions for human preimplantation embryos, which contribute to the birth of an estimated 1 million children every year (ESHRE 2023), remain unknown. Detailed disclosure of the concentrations of all components of commercial embryo culture media is required to facilitate evidence-based improvement of human embryo culture media, to potentially enhance IVF success rates and the health of children born after IVF.

To address this, other studies have previously measured the concentrations of various components in commercial human embryo culture media (Baltz, 2012; Morbeck *et al.*, 2014a, 2017; Tarahomi *et al.*, 2019) or protein supplements (Morbeck *et al.*, 2014b). However, some of these embryo culture media were analysed without supplementation of proteins (supplements) and did therefore not represent the environment that embryo culture media normally provide to human embryos. One study that did analyse supplemented embryo culture media included four continuous media (Morbeck *et al.*, 2017). We previously performed a composition analysis of 15 different ready-to-use commercial human embryo culture media and additionally studied the effects of culture medium storage and culture (Tarahomi *et al.*, 2019). In the present study, the medium composition analysis has been expanded to determine the concentrations of 40 components of 56 ready-to-use commercial human embryo culture media, including both sequential media and continuous media, along with 14 unsupplemented media and 10 supplements. This study, examining 80 different culture mediums and protein supplement samples, is the most comprehensive analysis of human embryo culture medium composition to date.

## Materials and methods

### Commercial human embryo culture media and protein supplements

Human embryo culture media and protein supplements from 11 different brands that were commercially available in the Netherlands between December 2019 and June 2020 were purchased from seven suppliers. Embryo culture media and protein supplements that became available on the market after this period and before the publication date were not included in the analysis. We included: 23 complete human embryo culture media (supplemented by the manufacturer): G-IVF PLUS, G-1 PLUS, G-2 PLUS, G-TL (Vitrolife, Sweden), Complete Early Cleavage Medium (with Dextran Serum Supplement; DSS), Complete MultiBlast Medium (with DSS), Continuous Single Culture Medium Complete (CSCM-C), Continuous Single Culture Medium-NX Complete (CSCM-NXC) (FUJIFILM Irvine Scientific, CA, USA), SAGE 1-Step HSA, SAGE Quinn’s Advantage Protein Plus Fertilization, ORIGIO Universal IVF Medium, ORIGIO Sequential Fert, ORIGIO Sequential Cleav, ORIGIO Sequential Blast, Global Total LP for Fertilization, Global Total LP, EmbryoGen, BlastGen (Cooper Surgical, CA, USA), Sydney IVF Fertilization Medium, Sydney IVF Cleavage Medium, Sydney IVF Blastocyst Medium (Cook Medical, IN, USA), GM501 (Gynemed, Germany) and GAIN Complete (FertiPro, Belgium); 14 unsupplemented human embryo culture media: G-IVF, G-1, G-2 (Vitrolife, Sweden), Early Cleavage Medium, MultiBlast Medium, Continuous Single Culture Medium, Continuous Single Culture Medium-NX (FUJIFILM Irvine Scientific, CA, USA), SAGE Quinn’s Advantage Fertilization Medium, SAGE Quinn’s Advantage Cleavage Medium, SAGE Quinn’s Advantage Blastocyst Medium, Global for Fertilization, Global (Cooper Surgical, CA, USA), IVC-TWO and IVC-THREE (InVitroCare Inc, MD, USA); and 10 protein supplements: Human Serum Albumin (HSA; Vitrolife, Sweden), recombinant human albumin supplement G-MM* (Vitrolife, Sweden), Human Serum Albumin (HSA; FUJIFILM Irvine Scientific, CA, USA), Serum Substitute Supplement (SSS*; FUJIFILM Irvine Scientific, CA, USA), Dextran Serum Supplement (DSS*; FUJIFILM Irvine Scientific, CA, USA), SAGE Quinn’s Advantage Human Serum Albumin (HSA; Cooper Surgical, CA, USA), SAGE Quinn’s Advantage Serum Protein Substitute (SPS*; Cooper Surgical, CA, USA), LifeGlobal Human Serum Albumin (HSA; Cooper Surgical, CA, USA), Human Serum Albumin (HSA; InVitroCare Inc, MD, USA), and Human Serum Albumin (HSA; Gynemed, Germany). Although the supplements with an asterisk (*) are not CE-marked and therefore not available for clinical use in Europe, we analysed their compositions in this study.

### Sample collection

Upon arrival, each complete medium (n = 23), unsupplemented medium (n = 14), and protein supplement (n = 10) was aliquoted in Eppendorf tubes, snap-frozen in liquid nitrogen, and stored at −80°C until composition analysis. Each unsupplemented medium (same bottle) was additionally supplemented with the corresponding protein supplement(s) according to the manufacturer’s instructions and then aliquoted, snap-frozen, and stored at −80°C until analysis. Global for Fertilization, Global, IVC-TWO, and IVC-THREE were supplemented with two different levels of HSA. Where Cooper Surgical’s instructions for Global media did not provide explicit supplementation guidance, clarification was sought, leading to the decision to analyse Global medium with both 5% and 10% LG HSA. For InVitroCare Inc media, we opted for the highest and lowest protein supplement percentages within the recommended supplementation range: 10–12% HSA for IVC-TWO and 12–15% HSA for IVC-THREE. In total, 80 different samples of ready-to-use embryo culture media (n = 56), unsupplemented embryo culture media (n = 14), and protein supplements (n = 10) were collected.

### Composition analysis

The concentration of 40 components was determined in all collected samples. Seven electrolytes (calcium (Ca^2+^), chloride (Cl^−^), iron (Fe^2+/3+^), potassium (K^+^), magnesium (Mg^2+^), sodium (Na^+^), and phosphate (${\text{PO}}_{4}^{3-}$)), glucose, immunoglobulins A, G, and M (IgA, IgG, and IgM), uric acid, alanine aminotransferase (ALAT), aspartate aminotransferase (ASAT), and albumin, as well as the total protein concentration, were quantified using a Cobas 8000 Analyser (Roche Diagnostics). Analysis of pyruvate, D- and L-lactate, D- and L-carnitine, and 21 amino acids (alanine, arginine, asparagine, aspartic acid, citrulline, glutamine, glutamic acid, glycine, histidine, isoleucine, leucine, lysine, methionine, ornithine, phenylalanine, proline, serine, threonine, tryptophan, tyrosine, and valine) was performed with Ultra-High Performance Liquid Chromatography-Mass Spectrometry (UPLC-MS/MS). All samples were thawed and prepared simultaneously for each analysis, and measurements were done by specialist technicians blinded to the names and brands of all embryo culture media and protein supplements.

## Results

**Figure 1.**
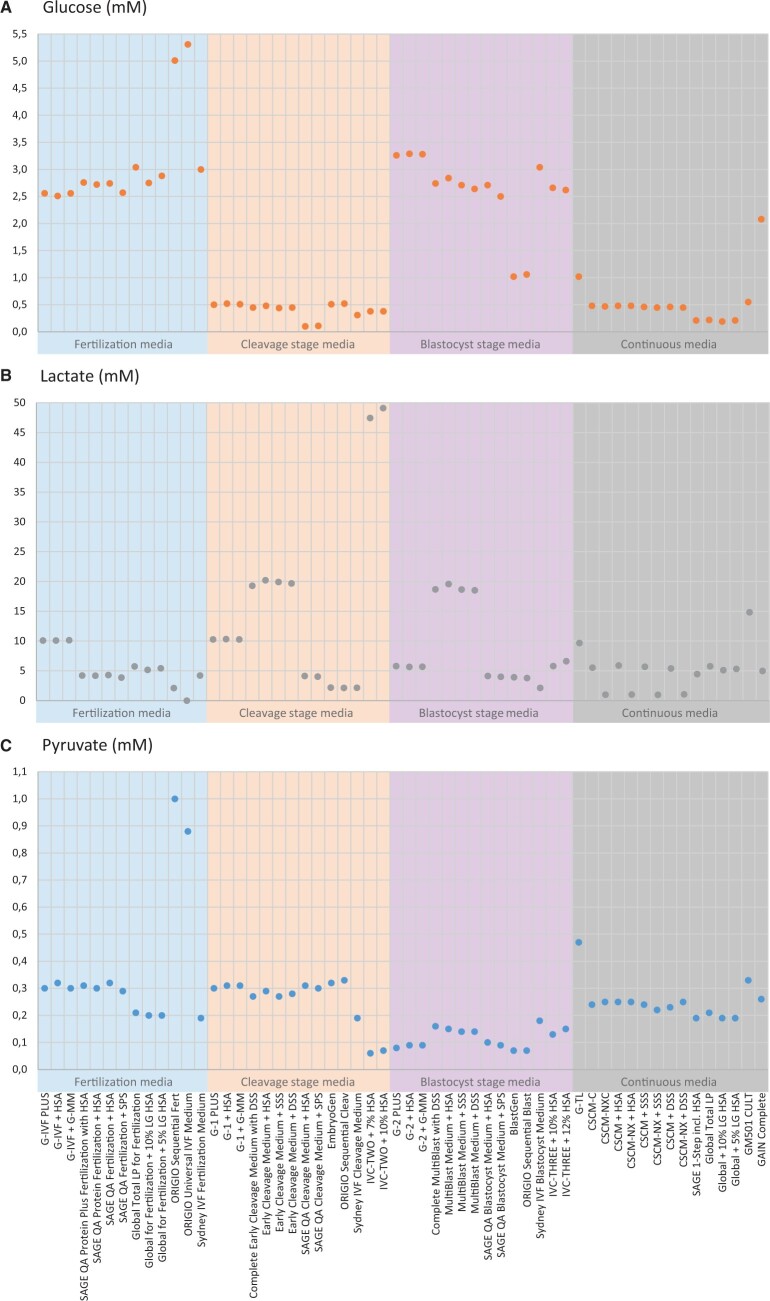
**Concentrations of energy sources (glucose, lactate, and pyruvate) determined in 56 ready-to-use (23 complete and 33 manually supplemented) commercial human embryo culture media.** (**A**) Glucose concentrations in mM. (**B**) Lactate concentrations in mM. Early Cleavage Media and MultiBlast Media contained both isomers of lactate: L-lactate (<50%) and the metabolically dead-end and thus toxic D-lactate (>50%) (see Supplementary Table S1a). All other dots represent the concentrations of 100% L-lactate in each human embryo culture medium. (**C**) Pyruvate concentrations in mM.

**Figure 2.**
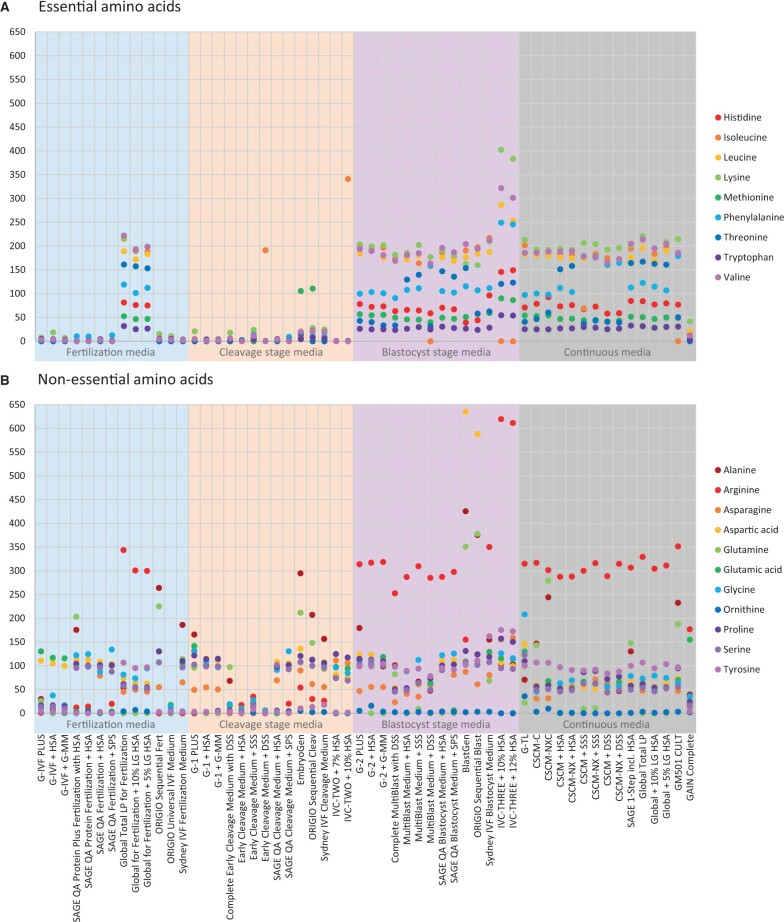
**Concentrations of amino acids determined in 56 ready-to-use (23 complete and 33 manually supplemented) commercial human embryo culture media.** (**A**) Essential amino acid concentrations in μM. (**B**) Non-essential amino acid concentrations in μM. Citrulline was not included in this graph, as it was not detected in any of the culture media. Glycine concentrations of >3000 μM are shown in Supplementary Fig. S1B.

**Figure 3.**
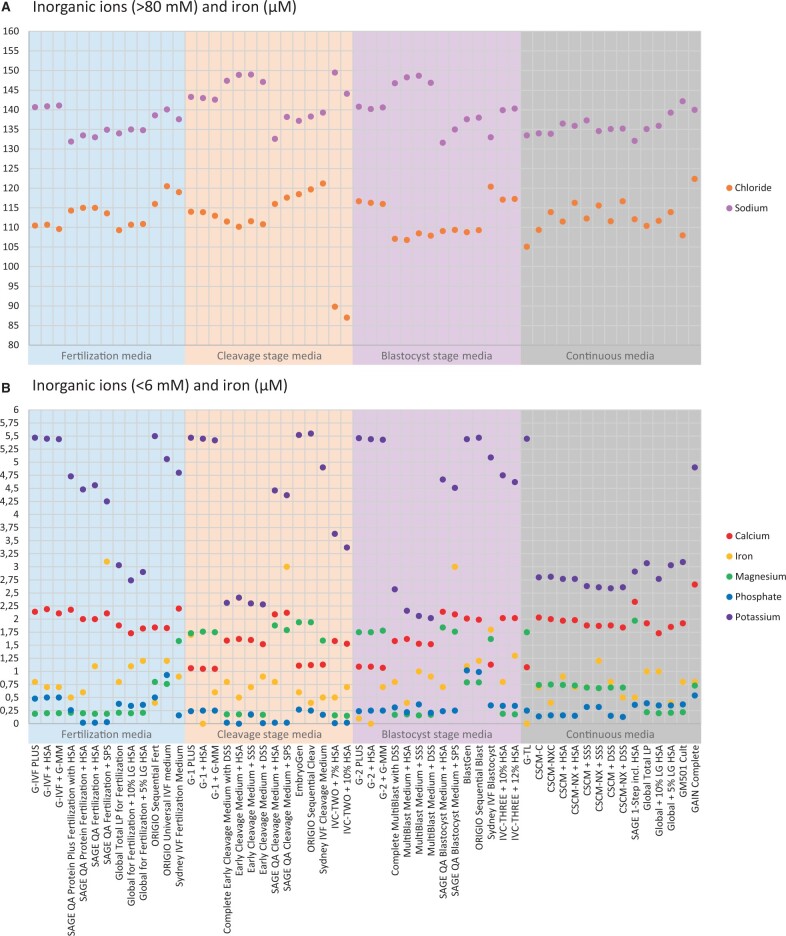
**Concentrations of inorganic ions (chloride, sodium, calcium, magnesium, phosphate, potassium) and iron determined in 56 ready-to-use (23 complete and 33 manually supplemented) commercial human embryo culture media.** (**A**) Chloride and sodium concentrations (in mM) (**B**) Calcium, magnesium, phosphate, potassium (all in mM), and iron (in μM) concentrations.

Concentrations of 40 components were determined in 56 samples of complete human embryo culture media (n = 23) and manually supplemented human embryo culture media (n = 33; together also referred to as ready-to-use embryo culture media) (Figs 1, 2, and 3 and Supplementary Fig. S1 and Supplementary Table S1a) and in unsupplemented human embryo culture media (n = 14) and protein supplements (n = 10) separately (Supplementary Figs S2, S3, S4, and S5 and Supplementary Table S1b and c).

### Energy sources

*Glucose* was detected in all ready-to-use embryo culture media (Fig. 1A and Supplementary Table S1a). Glucose concentrations followed a high-low-high pattern in sequential media systems: most fertilization media contained 2.5–3.0 mM, most cleavage stage media contained only 0.5 mM or less, and most blastocyst media contained 2.5–3.3 mM. Glucose concentrations in ORIGIO media (including BlastGen) deviated from the general pattern (Fig. 1A and Supplementary Table S1a). Glucose concentrations in continuous media were mainly relatively low and similar to the glucose concentrations in cleavage stage media (0.5 mM or below). Three continuous media deviated from this general pattern: G-TL had a glucose concentration of 1.0 mM, GAIN Complete contained over 2.1 mM glucose, and the 0.2 mM glucose in SAGE-1-Step was higher compared to SAGE Quinn’s Advantage Cleavage Medium.

*Lactate* (L-lactate) was detected in all ready-to-use embryo culture media, except ORIGIO Universal IVF medium (Fig. 1B and Supplementary Table S1a). We analysed both isoforms, L- and D-lactate, and confirmed that Early Cleavage and MultiBlast media from FUJIFILM Irvine Scientific contained both L- and D-lactate, in line with our previous findings (Tarahomi *et al.*, 2019). Overall absolute lactate concentrations, as well as lactate concentration patterns in sequential media systems, varied among embryo culture media from all brands. For example, although lactate concentrations in cleavage stage media from Vitrolife, SAGE Quinn’s Advantage, and ORIGIO were the same compared to the corresponding fertilization media, absolute lactate concentrations differed notably between these brands: 10.1 mM in G-IVF and 10.2 mM in G-1, 3.9–4.3 mM in SAGE Quinn’s Advantage Fertilization and Cleavage Media and 2.1 mM in ORIGIO Sequential Fert and Cleav. The lactate concentration was lower (5.7 mM) in G-2, similar (4.1 mM) in SAGE Quinn’s Advantage Blastocyst Medium and higher (3.8 mM) in ORIGIO Sequential Blast. ORIGIO’s EmbryoGen and BlastGen aligned with the concentrations of ORIGIO Sequential Cleav and Blast. A different pattern was seen in Sydney Sequential media: 4.2 mM lactate in Sydney Sequential Fertilization Medium, a lower lactate concentration of 2.2 mM in Sydney Sequential Cleavage Medium and a similar concentration of 2.1 mM in Sydney Sequential Blastocyst Medium. Both Early Cleavage and MultiBlast media had a relatively high lactate concentration of around 20 mM. IVC-TWO medium samples contained the highest lactate concentrations: almost 50 mM. IVC-THREE medium contained a lower concentration of ∼6 mM. Vitrolife, SAGE Quinn’s Advantage, and Global continuous media mirrored lactate concentrations of the sequential media. Additionally, FUJIFILM Irvine Scientific had a low lactate variant of CSCM(-C): CSCM-NX(C), with a lactate concentration of ∼1 mM.

*Pyruvate* was detected in all ready-to-use embryo culture media (Fig. 1C and Supplementary Table S1a). Most fertilization and cleavage stage media contained a pyruvate concentration between 0.2 and 0.3 mM. However, ORIGIO Sequential Fert medium and ORIGIO Universal IVF medium had a 3-fold higher pyruvate concentration of respectively 1.0 and 0.9 mM, and IVC-TWO medium had a relatively low pyruvate concentration of <0.1 mM. Blastocyst media from most medium manufacturers contained a lower pyruvate concentration of 0.1–0.2 mM. In contrast, although the pyruvate concentration in IVC-THREE medium was in the same range as other blastocyst media, it was twice as high as the relatively low pyruvate concentration in IVC-TWO medium. The concentration pyruvate in Sydney IVF Blastocyst medium remained the same compared to the fertilization and cleavage stage media. Continuous media had pyruvate concentrations similar to fertilization and cleavage stage media of the same brand (0.2–0.3 mM). The concentration pyruvate in G-TL was notably higher in comparison with other continuous media and the Vitrolife sequential media.

*Carnitine* was undetectable in all ready-to-use embryo culture media, except for SAGE Quinn’s Advantage sequential media supplemented with SPS, which contained >0.2 μM L-carnitine (Supplementary Fig. S1A and Supplementary Table S1a). Although L-carnitine was identified in 8 out of the 10 analysed protein supplements (Supplementary Fig. S3B and Supplementary Table S1c), only the notably higher, but still relatively low, concentration of 2.5 μM in SPS resulted in these low, but detectable carnitine levels in the ready-to-use media.

### Amino acids

Although amino acid concentrations varied, a clear general pattern was found in the concentrations of 11 amino acids in almost all ready-to-use embryo culture media.

Whereas the concentrations of histidine, isoleucine, leucine, lysine, methionine, phenylalanine, threonine, tryptophan, and valine, all *essential amino acids*, were zero or low in fertilization and cleavage stage media, they were present in higher levels in almost all blastocyst media (Fig. 2A and Supplementary Table S1a). In contrast, Global (Protein) Fertilization media showed essential amino acid concentrations that were similar to the concentrations in blastocyst stage media of other brands. IVC-THREE medium samples had consistently elevated concentrations of essential amino acids compared to blastocyst media from other brands. Most manufacturers mimicked the essential amino acid concentrations of the blastocyst media in the continuous media. Concentrations of all essential amino acids in GAIN Complete medium were lower compared to all other continuous media.

In contrast, concentrations of the *non-essential amino acids* in ready-to-use embryo culture media did not show a clear pattern (Fig. 2B and Supplementary Table S1a). Most concentrations of alanine, arginine, asparagine, aspartic acid, glutamine, glutamic acid, glycine, proline, serine, and tyrosine were roughly between 50 and 150 μM in all ready-to-use media. However, generally lower concentrations were seen in G-IVF media (≤30 μM, except glutamic acid and aspartic acid), ORIGIO Universal IVF Medium (all ≤18 μM) and Early Cleavage Medium and MultiBlast Medium (all ≤27 μM). In most continuous media, non-essential amino acid concentrations were roughly 50 μM, slightly lower than in sequential media. Exceptions were slightly higher overall concentrations in G-TL (up to 208 μM) and slightly lower overall concentrations in GAIN Complete (all ≤39 μM, except glutamic acid). Additionally, concentrations of citrulline and ornithine, two non-essential amino acids not involved in protein synthesis that we also analysed, were respectively undetected and present in concentrations close to zero in all analysed samples.

Interestingly, arginine and tyrosine concentration patterns appeared to match the general pattern of essential amino acid concentrations: low in fertilization and cleavage stage media, higher in blastocyst and continuous media. Arginine and tyrosine concentrations in Global for Fertilization media were also higher and an exception from the general pattern, in line with essential amino acid concentrations (Fig. 2A and Supplementary Table S1a). Also remarkably, glycine concentrations in EmbryoGen and BlastGen, ORIGIO Sequential Fert, Cleav and Blast and Sydney IVF Fertilization, Cleavage and Blastocyst Media were up to 12.5 times higher than the average glycine concentration in other embryo culture media (Fig. 2B, Supplementary Fig. S1B and Supplementary Table S1a). Furthermore, glutamine and alanine concentrations were notably higher in SAGE Quinn’s Advantage Protein Plus Fertilization with HSA, EmbryoGen and BlastGen, ORIGIO Sequential media, CSCM-C and CSCM-NXC, SAGE-1-Step incl. HSA and GM501 CULT. Interestingly, glutamine was present in 20 out of 23 complete embryo culture media with concentrations from 25 to 378 μM, where it was absent or present up to 2 μM in their manually supplemented equivalent (Fig. 2B and Supplementary Table S1a). Embryo culture media from FUJIFILM Irvine scientific that were manually supplemented with SSS were an exception and did contain ∼10 μM glutamine, likely resulting from the higher glutamine concentration of 100 μM in the supplement SSS (Fig. 2B, Supplementary Fig. S4B and Supplementary Table S1a and c).

### Inorganic ions and iron

Calcium, chloride, iron, magnesium, phosphate, potassium, and sodium were all available in almost all ready-to-use embryo culture media (Fig. 3A and B and Supplementary Table S1a). *Magnesium* concentrations exhibited two different patterns in sequential media systems. Low concentrations (0.8 mM or below) were consistently maintained for all developmental stages in embryo culture media from Global, FUJIFILM Irvine Scientific, and InVitroCare Inc. Contrastingly, embryo culture media from Vitrolife, SAGE Quinn’s Advantage, and Sydney demonstrated higher magnesium concentrations in cleavage and blastocyst stage media, as well as continuous media (Fig. 3B and Supplementary Table S1a). Differently, ORIGIO media (including EmbryoGen and BlastGen) showed a high-low-high pattern (0.8 mM in the fertilization stage, 1.9 in the cleavage stage, 0.8 in the blastocyst stage). In continuous media, magnesium concentrations were highest in G-TL (1.8 mM) and SAGE-1-Step (2.0 mM). *Potassium* concentrations were generally constant in all sequential and continuous media from the same brand, but clearly varied in absolute concentrations between brands (Fig. 3B and Supplementary Table S1a). Where continuous media from other brands contained a potassium concentration between 2.6 and 3.1 mM, higher concentrations were found in G-TL (5.5 mM) and GAIN Complete (3.9 mM). *Calcium* concentrations (1.1–2.7 mM) varied among the different brands in cleavage stage and blastocyst stage media and in continuous media (Fig. 3B and Supplementary Table S1a). *Phosphate* concentrations were 0.5 mM or below in most embryo culture media, except in ORIGIO Universal IVF medium (0.9 mM), BlastGen (1.0 mM), and ORIGIO Sequential Blast (1.0 mM) (Fig. 3B and Supplementary Table S1a). *Iron* concentrations varied between 0 and 1.8 µM in most samples, but was 3.0–3.1 µM in all SAGE Quinn’s Advantage media supplemented with SPS (Fig. 3B and Supplementary Table S1a). Both *chloride* (105.1–122.4 mM) and *sodium* (131.6–150.4 mM) concentrations were similar in all media from different brands, except the chloride concentrations of below 90 mM in IVC-TWO samples (Fig. 3A and Supplementary Table S1a).

### Other components

Other components that were analysed in ready-to-use embryo culture media were uric acid and immunoglobulins IgA, IgG, and IgM, which were not detected or at a level that was almost zero, as well as albumin and total protein, which were generally a bit below 5 or 10 mM (albumin) and around 5 or 10 mM of total protein, and ALAT and ASAT, which were present in almost all samples (ALAT up to 4.3 U/l and ASAT up to 4.4 U/l) (Supplementary Table S1a–c). ALAT and ASAT were additionally analysed, since we previously found these liver enzymes to be present and affecting the composition of embryo culture media during embryo culture (Tarahomi *et al.*, 2019).

## Discussion

We determined the composition of the commercial human embryo culture media available that we could purchase in the Netherlands and provided a comprehensive report on the concentrations of 40 components in 56 ready-to-use human embryo culture media.

This study is unique in its analysis of embryo culture media for all sequential stages and the comparison with continuous media, together representing the most comprehensive analysis of ready-to-use embryo culture media composition within a single experiment. Additional analyses of unsupplemented media and protein supplements for the same components enable tracing of each components’ origin. We limited our purchase to one lot number of all embryo culture media and protein supplements. Reliability of the measurements was confirmed by semi-duplicates within our 80 samples: different samples of the same culture medium before and after supplementation with a protein supplement. Our results were also validated by partial overlap with previous studies that analysed 32–39 components in commercial embryo culture media, as the concentrations of many overlapping components are similar to those found by Morbeck *et al.* (2014a, 2017) and Tarahomi *et al.* (2019). A limitation of this study is that the embryo culture media will likely contain more components than measured in this study as we used a targeted approach. Part of the unidentified components could originate from the protein supplements added to the embryo culture media, which are the most undefined and variable ‘component’ of ready-to-use human embryo culture media (Dyrlund *et al.*, 2014). We did not analyse the presence and concentrations of vitamins, growth factors, or anti-oxidants, some of which are expected to be present in at least some of the included embryo culture media as they are mentioned on the ingredient list of several media.

Our results showed that no two human embryo culture media were the same. Although similarities were found, the concentrations of several components varied between all embryo culture media. Importantly, current concentrations of components seem to be based on limited available evidence. Below we discuss the most notable differences in concentrations of some of the components we found in commercial human embryo culture media, with the perspective of the often limited evidence for these concentrations in the scientific literature.

### Energy sources

*Glucose* concentrations in all cleavage stage media were ∼0.5 mM or lower, which is mirrored in continuous media and notably lower than in fertilization and blastocyst stage media (Fig. 1A and Supplementary Table S1a). This pattern likely originates from the observation that hamster embryos failed to develop beyond the two-cell stage in the presence of glucose and phosphate, which may have led to cautiousness in adding glucose to cleavage stage media (Yanagimachi and Chang, 1964; Schini and Bavister, 1988). Furthermore, measurements of six midcycle human oviduct fluid samples later confirmed that a low glucose concentration of 0.5 mM seems appropriate for cleavage stage culture (Gardner *et al.*, 1996). However, experimental studies in the late nineties and early 2000s showing embryo development into blastocysts in embryo culture media with a higher glucose concentration in combination with a different medium composition, did not receive adequate attention (for review, see Summers and Biggers, 2003). Although since then, it was generally accepted that glucose concentrations do not absolutely inhibit the first cleavages of preimplantation embryos and generally discouraged to minimize glucose concentrations in cleavage stage media, medium manufacturers have continued to maintain low glucose concentrations to this day.

A recent study that also analysed midcycle human oviduct fluid (n = 21), found an average *in vivo* glucose concentration of ∼3.4 mM (Utsunomiya *et al.*, 2022). Additionally, our group measured an elevated glucose concentration of 5.1 mM in 22 samples of human uterine fluid from healthy, fertile women 3 days after a positive LH test or ovum pick-up (Tarahomi *et al.*, 2024). Although the first study included subfertile women, these findings suggest that the physiological glucose concentration is comparable to or higher than commonly present in fertilization and blastocyst stage media and that lower glucose concentrations should be considered as non-physiological. Elevating the glucose concentrations in cleavage stage and continuous media according to the *in vivo* evidence, requires improving the concentrations of other medium components for successful support of blastocyst development (Gardner and Lane, 1996). Particularly continuous media could significantly benefit from an effort in optimizing medium composition by aligning the glucose concentration with *in vivo* levels, as an adequate amount of glucose is crucial for energy provision after compaction (Gardner, 2008). All in all, the discrepancies in reported glucose concentrations *in vivo* and what we now found *in vitro* deserve more attention.

*Lactate* concentrations varied widely in all embryo culture media and no consistent pattern was observed across the sequential media systems from all brands (Fig. 1B and Supplementary Table S1a). The variation highlights the absence of a general rationale on the optimal lactate concentration in culture media for human preimplantation embryos. This is concerning, as lactate concentrations have been shown to interfere with pyruvate oxidation, the primary energy source for zygotes and cleavage stage embryos (Lane and Gardner, 2000). For example, the lactate concentration of almost 50 mM found in IVC-TWO medium may hinder embryos from effectively utilizing pyruvate for energy during the cleavage stage. In mice, a physiological lactate concentration of 5 mM or below appeared to be acceptable, as pyruvate uptake in mouse cleavage stage embryos was shown to be maintained (Lane and Gardner, 2000). Although the physiological lactate concentration in human oviducts was long considered to be around 10.5 mM based on analysis of six samples of midcycle human oviduct fluid (Gardner *et al.*, 1996), a recent study found a lower lactate concentration of 5.1 mM in midcycle oviduct fluid from 21 women, supporting a lactate concentration of around 5 mM in cleavage stage media (Utsunomiya *et al.*, 2022). Additionally, our group found a lactate concentration of 6.6 mM in 22 samples of human uterine fluid 3 days after a positive LH test or ovum pick-up (Tarahomi *et al.*, 2024). These recent *in vivo* findings suggest that a lactate concentration in the range of 5–7 mM would be considerable for human preimplantation embryos until the blastocyst stage. However, metabolic balance is suggested to depend on the ratio between lactate concentration and pyruvate concentration, rather than absolute concentrations alone (Morbeck *et al.*, 2014a). Furthermore, where all other embryo culture media specifically contained L-lactate, over 50% of the total lactate measured in Early Cleavage and MultiBlast media from FUJIFILM Irvine Scientific was identified as the metabolically dead-end and thus toxic D-lactate, as was also identified by us previously (Tarahomi *et al.*, 2019). Interestingly, between ordering the media and publication of this study, Early Cleavage and MultiBlast medium have been taken off the market and FUJIFILM Irvine Scientific now only provides continuous media: CSCM(-C) and low lactate media CSCM-NX(C).

*Pyruvate* concentrations show a trend with higher concentrations in fertilization media and cleavage stage media (both 0.2–0.3 mM), and lower concentrations in blastocyst stage media (roughly around 0.1 mM) from most brands (Fig. 1C and Supplementary Table S1a). To a certain extent, these concentrations resemble the concentrations found in midcycle human oviduct fluid (0.32 mM; n = 6) and human uterine fluid throughout the menstrual cycle (0.10 mM; n = 15) (Gardner *et al.*, 1996). More recent *in vivo* analyses determined pyruvate concentrations of 0.209 mM in midcycle oviduct fluid (n = 21) and 0.106 mM in the luteal phase (n = 28) (Utsunomiya *et al.*, 2022), and 0.08 mM in human uterine fluid (n = 22) (Tarahomi *et al.*, 2024). The lower pyruvate concentrations in most blastocyst media compared to fertilization and cleavage stage media align with the general idea that pyruvate is the preferred energy source for preimplantation embryos until embryonic genome activation or the blastocyst stage (Gardner, 2008). In contrast, the consistent pyruvate concentrations in Sydney IVF sequential media align with the suggestion that pyruvate uptake by preimplantation embryos remains constant, even in the blastocyst stage, and dependent on the medium composition particularly the lactate concentration in the medium (Lane and Gardner, 2000; Lee *et al.*, 2022). The rationale for the elevated pyruvate concentrations in ORIGIO fertilization media and continuous medium G-TL is unclear.

The optimal ratio of lactate to pyruvate concentrations in the human preimplantation embryo environment is unknown. In blood, a lactate-pyruvate ratio between 10 and 20 is considered normal. The *in vivo* findings from 1996 indicated lactate-pyruvate ratios of ∼33 in human oviduct fluid and 59 in human uterine fluid (Gardner *et al.*, 1996). The more recent *in vivo* findings, however, suggest ratios of 25–30 in oviduct fluid and about 83 in uterine fluid (Utsunomiya *et al.*, 2022; Tarahomi *et al.*, 2024). In contrast, our analysis revealed a high variability in lactate-pyruvate ratios within embryo culture media, ranging from 0 to 719, confirming previous observations of high variability in lactate-pyruvate ratios in embryo culture media (Morbeck *et al.*, 2014a). Since the lactate-pyruvate ratio in the embryo environment directly influences the metabolic activity of an embryo, affecting the amount of energy available for embryo development, further research into the lactate-pyruvate ratio is important for future culture medium development (Zander-Fox and Lane, 2012).

*Carnitine* was included in our analysis in light of the growing interest in carnitine as a potential component in embryo culture media due to its role in fatty acid metabolism and energy production to maintain metabolic homeostasis and its roles as scavenger of reactive oxygen species (ROS) and cryoprotector (for review, see Placidi *et al.*, 2022). Experimental studies have shown that the addition of acetyl-L-carnitine to embryo culture media improves oocyte and embryo development and cryotolerance of mouse, porcine, bovine, and human preimplantation embryos (Truong *et al.*, 2016; Lowe *et al.*, 2017; Truong and Gardner, 2017, 2020; Zolini *et al.*, 2019; Gardner *et al.*, 2020). Although carnitine was not detected in most ready-to-use embryo culture media included in this study (Supplementary Fig. S1A and Table S1A), it may be included in future versions of these media or new embryo culture media. Whether carnitine is present in human oviduct and uterine fluid is unknown.

### Amino acids

*Essential amino acids* were absent or present at low concentrations in fertilization and cleavage stage media, but consistently appeared in higher concentrations in blastocyst and continuous media from most brands (Fig. 2A and Supplementary Table S1a). In contrast, *non-essential amino acids* were generally present, but varied in their absolute concentrations among the different types of embryo culture media and brands (Fig. 2B and Supplementary Table S1a). These patterns may reflect the findings from an experimental study showing that mouse embryo development and viability are greatest in the consistent presence of non-essential amino acids and glutamine and when essential amino acids are exclusively added after the eight-cell stage (Lane and Gardner, 1997). The contrasting presence of essential amino acids in Global for Fertilization media may be influenced by another experimental study with mouse embryos suggesting that zygotes have enhanced developmental potential when produced in mKSOM supplemented with 19 amino acids (Summers *et al.*, 2000). *In vivo* studies showed that both essential and non-essential amino acids are present in midcycle human oviduct and uterine fluid (Kermack *et al.*, 2015; Utsunomiya *et al.*, 2022). However, absolute concentrations of both essential amino acids and non-essential amino acids in these *in vivo* fluids differ from the concentrations measured in the embryo culture media. The *in vivo* evidence suggests that the absence or low concentrations of essential amino acids in media for the early stages of human embryo culture are non-physiological and might represent an adaptation to support human embryo development *in vitro* in a suboptimal medium composition.

Glycine concentrations in three sequential media systems were markedly higher than in all other embryo culture media and exceeded 3000 μM (Supplementary Fig. S1B and Supplementary Table S1a). Elevation of these glycine concentrations could be attributed to its role as an organic osmolyte, although a concentration of 1000 μM glycine was earlier found to be maximally effective for mouse preimplantation embryos (Baltz, 2012). This was supported by enhanced development of early- and late-stage bovine embryos in the presence of 1100 μM glycine (Herrick *et al.*, 2016). Interestingly, the physiological glycine concentration in (early luteal phase) human uterine fluid was also found to be around 1000 μM (Kermack *et al.*, 2015; Tarahomi *et al.*, 2024) and the average glycine concentration in 21 samples of midcycle oviduct fluid was demonstrated to be 462 μM (Utsunomiya *et al.*, 2022). Given that the *in vivo* glycine concentration appears to be around 1000 μM in human uterine fluid and is notably higher in human oviduct fluid than in most human embryo culture media, and considering that 1000 μM is the optimal glycine concentration for mouse and bovine embryos, investigating the effect of increasing the glycine concentration up to 1000 μM in human embryo culture media might be of interest.

The reasons for substantial glutamine concentration discrepancies between complete embryo culture media, in which the concentration is notably high, and the same media after manual supplementation, in which glutamine is generally absent, remain unclear (Fig. 2B and Supplementary Table S1a). *In vivo* studies reported different glutamine concentrations: 130 μM in midcycle human oviduct fluid (n = 21), 426 μM in human uterine fluid throughout the cycle (n = 56) and 227 μM in early luteal human uterine fluid (n = 22) (Kermack *et al.*, 2015; Utsunomiya *et al.*, 2022; Tarahomi *et al.*, 2024). Glutamine’s potential benefits for embryo development are linked to its roles as organic osmolyte or an extra carbon source for preimplantation embryos. The presence of 1000 μM glutamine was found to be optimal in overcoming the 2-cell block in mouse embryos (Chatot *et al.*, 1989; Lawitts and Biggers, 1992), and that concentration was later applied in potassium simplex optimization medium (KSOM) and Vitrolife sequential media (Summers and Biggers, 2003). However, free glutamine then appeared to be detrimental for embryo development due to ammonium accumulation (Lane and Gardner, 1994), resulting in the addition of a stable dimer of alanyl- or glycyl-L-glutamine to embryo culture media to prevent this quick ammonium build-up (Lane *et al.*, 2001). Elevated alanine concentrations in conjunction with higher glutamine concentrations suggest the addition of alanyl-L-glutamine to the complete embryo culture media included in our analysis.

### Inorganic ions and iron

The concentrations of inorganic ions determined in the embryo culture media were similar to the findings of Morbeck *et al.* (2014a, 2017) and Tarahomi *et al.* (2019). The biggest differences in concentrations of these ions were seen for calcium, magnesium, and potassium (Fig. 3B and Supplementary Table S1a). Calcium concentrations (1.1–2.7 mM) and magnesium concentrations (0.2–2.0 mM) identified in the embryo culture media are comparable to the average concentrations found in midcycle to luteal phase human oviduct fluid: in midcycle 1.3 mM calcium (n = 21) and in luteal phase 1.1 mM calcium (n = 21) and 1.1 mM calcium (n = 7), and in midcycle 0.5 mM magnesium (n = 21) and in luteal phase 0.6 mM magnesium (n = 21) and 1.4 mM magnesium (n = 7) (Borland *et al.*, 1980; Utsunomiya *et al.*, 2022). Potassium concentrations in all embryo culture media did not match the average concentrations determined in midcycle luteal phase human oviduct fluid (midcycle 15.8 mM (n = 21), luteal phase 15.8 mM (n = 21) and 21.2 mM (n = 7)) (Borland *et al.*, 1980; Utsunomiya *et al.*, 2022). An experimental mouse embryo study suggested that a potassium concentration of 25 mM, which is comparable to the 26.5 mM identified in proestrous mouse ampullary fluid (Borland *et al.*, 1977), is optimal for mouse embryo culture as this concentration led to the best embryo development and highest implantation rate. However, a concentration of 10 mM, closer to proestrous mouse uterine fluid concentrations (14.1 mM), seemed to better support blastocyst formation (Roblero and Riffo, 1986). Global media contained potassium concentrations comparable to the concentration in KSOM (2.5 mM) (Erbach *et al.*, 1994). Research on the effect of resembling a physiological potassium concentration in culture media for human embryo culture seems warranted, as evidence for the low potassium concentrations in human embryo culture media is absent. For further discussion of the concentrations of calcium, magnesium, and potassium in commercial embryo culture media, we refer to Morbeck *et al.* (2017).

Iron concentrations were highest in media supplemented with SPS, similar to previous measurements (Morbeck *et al.*, 2014b). We did not analyse other metals that are known to be present in embryo culture media, like aluminium, chromium, and manganese (Morbeck *et al.*, 2014a).

## Conclusion

In conclusion, this study has highlighted the persistent uncertainty about the optimal composition of culture media for human preimplantation embryos. This was illustrated by the fact that none of the embryo culture media from different manufacturers were identical. In fact, one supplier, Cooper surgical, offers embryo culture media from four different brands with significantly different compositions. If the ideal composition were known, marketing multiple embryo culture media with varying compositions by a single supplier would seem inconceivable. Despite shared trends among various embryo culture media, the current compositions of commercial embryo culture media appear to be based on limited available evidence. Further research aimed at elucidating the natural *in vivo* environment of human preimplantation embryos may provide critical insights for optimization of the composition of culture media for *in vitro* culture of human preimplantation embryos. Outcomes of this type of research have the potential to enhance IVF success rates and improve child health outcomes after IVF. Complete disclosure of the current compositions of commercial human embryo culture media by manufacturers, along with ongoing transparency in the development and improvement of these media, is essential for safe and effective optimization of the *in vitro* environment of human preimplantation embryos.

## Supplementary Material

deae248_Supplementary_Table_S1a,b,c

deae248_Supplementary_Figure_S1

deae248_Supplementary_Figure_S2

deae248_Supplementary_Figure_S3

deae248_Supplementary_Figure_S4

deae248_Supplementary_Figure_S5

## Data Availability

The data underlying this article are available in the article and in the online supplementary material.
