# Supplementary material for: The composition of commercially available human embryo culture media
Source: Hum Reprod. 2024 Nov 25;40(1):30–40. doi: 10.1093/humrep/deae248 (PMC11700899; doi:10.1093/humrep/deae248)
Supplement: deae248_Supplementary_Figure_S1 [file deae248_supplementary_figure_s1.pdf]

OXFORD
human reproduction

Human Reproduction, 2024, 1–1
https://doi.org/10.1093/humrep/deae248
Supplementary Data

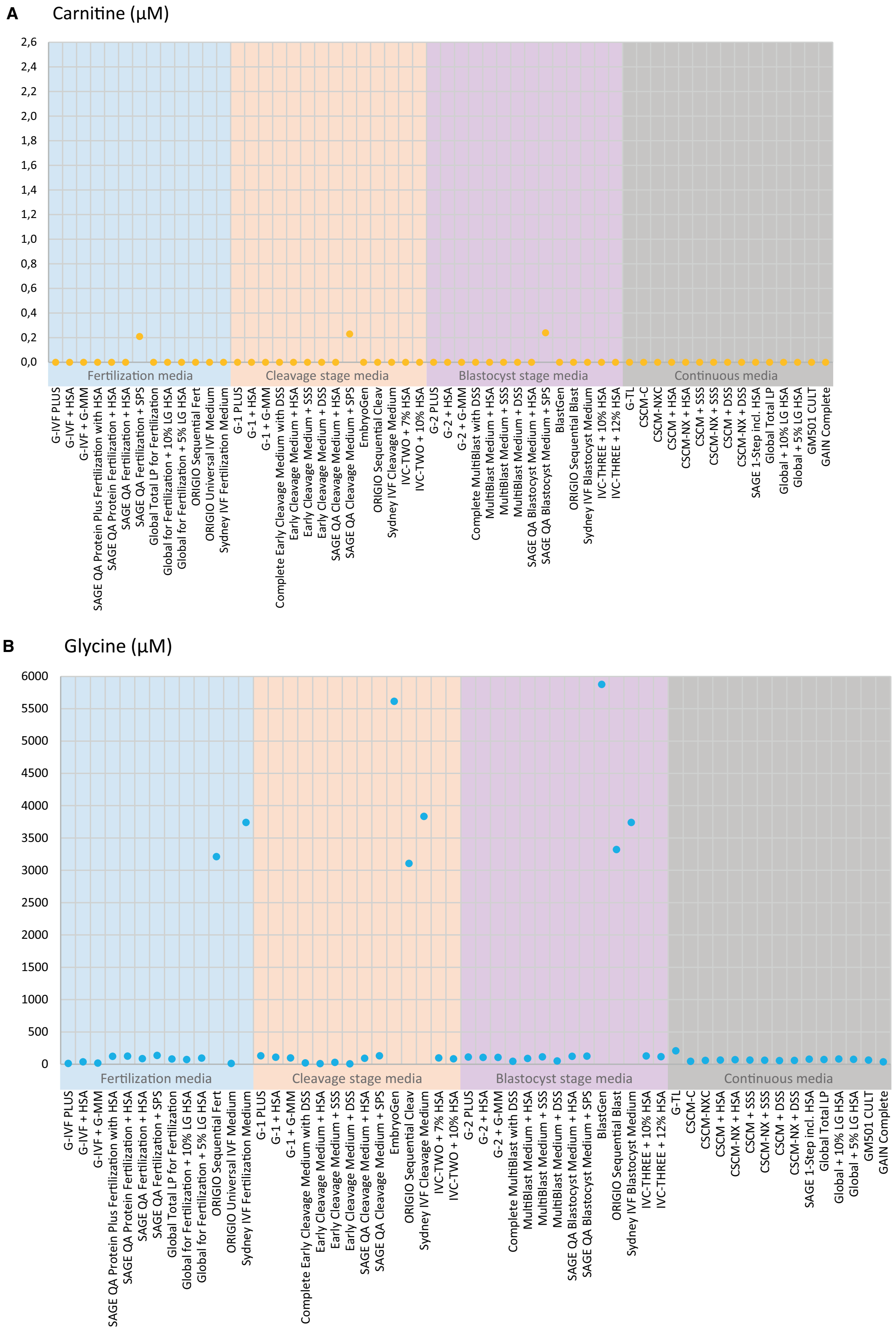

Supplementary Figure S1. Concentrations of energy sources (carnitine and glycine) determined in 56 ready-to-use (23 complete and 33 manually supplemented) commercial human embryo culture media. (**A**) Carnitine (L-carnitine) concentrations in µM. (**B**) Glycine concentrations in µM.
