# Supplementary material for: The composition of commercially available human embryo culture media
Source: Hum Reprod. 2024 Nov 25;40(1):30–40. doi: 10.1093/humrep/deae248 (PMC11700899; doi:10.1093/humrep/deae248)
Supplement: deae248_Supplementary_Figure_S2 [file deae248_supplementary_figure_s2.pdf]

OXFORD
human reproduction
Human Reproduction, 2024, 1–1
https://doi.org/10.1093/humrep/deae248
Supplementary Data

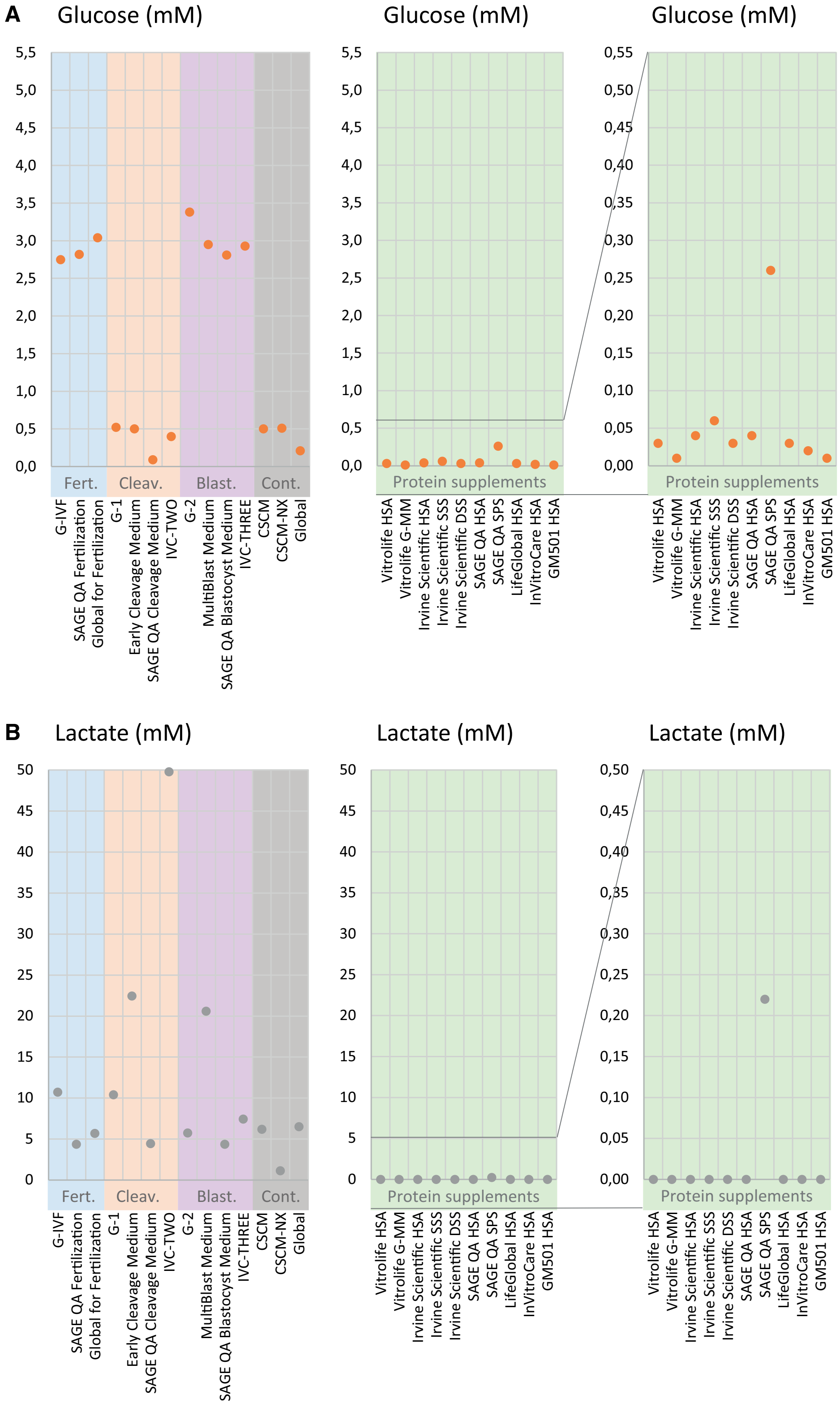

Supplementary Figure S2. Concentrations of energy sources (glucose and lactate) determined in 14 unsupplemented commercial human embryo culture media and 10 protein supplements. (**A**) Glucose concentrations in mM. (**B**) Lactate concentrations in mM. Early Cleavage Medium and MultiBlast Medium contained both isomers of lactate: L-lactate (<50%) and the metabolically dead-end and thus toxic D-lactate (>50%) (see Supplementary Table S1a). All other dots represent the concentrations of 100% L-lactate in each human embryo culture medium.
