# Supplementary material for: The composition of commercially available human embryo culture media
Source: Hum Reprod. 2024 Nov 25;40(1):30–40. doi: 10.1093/humrep/deae248 (PMC11700899; doi:10.1093/humrep/deae248)
Supplement: deae248_Supplementary_Figure_S3 [file deae248_supplementary_figure_s3.pdf]

human reproduction

Human Reproduction, 2024, 1–1
https://doi.org/10.1093/humrep/deae248
Supplementary Data

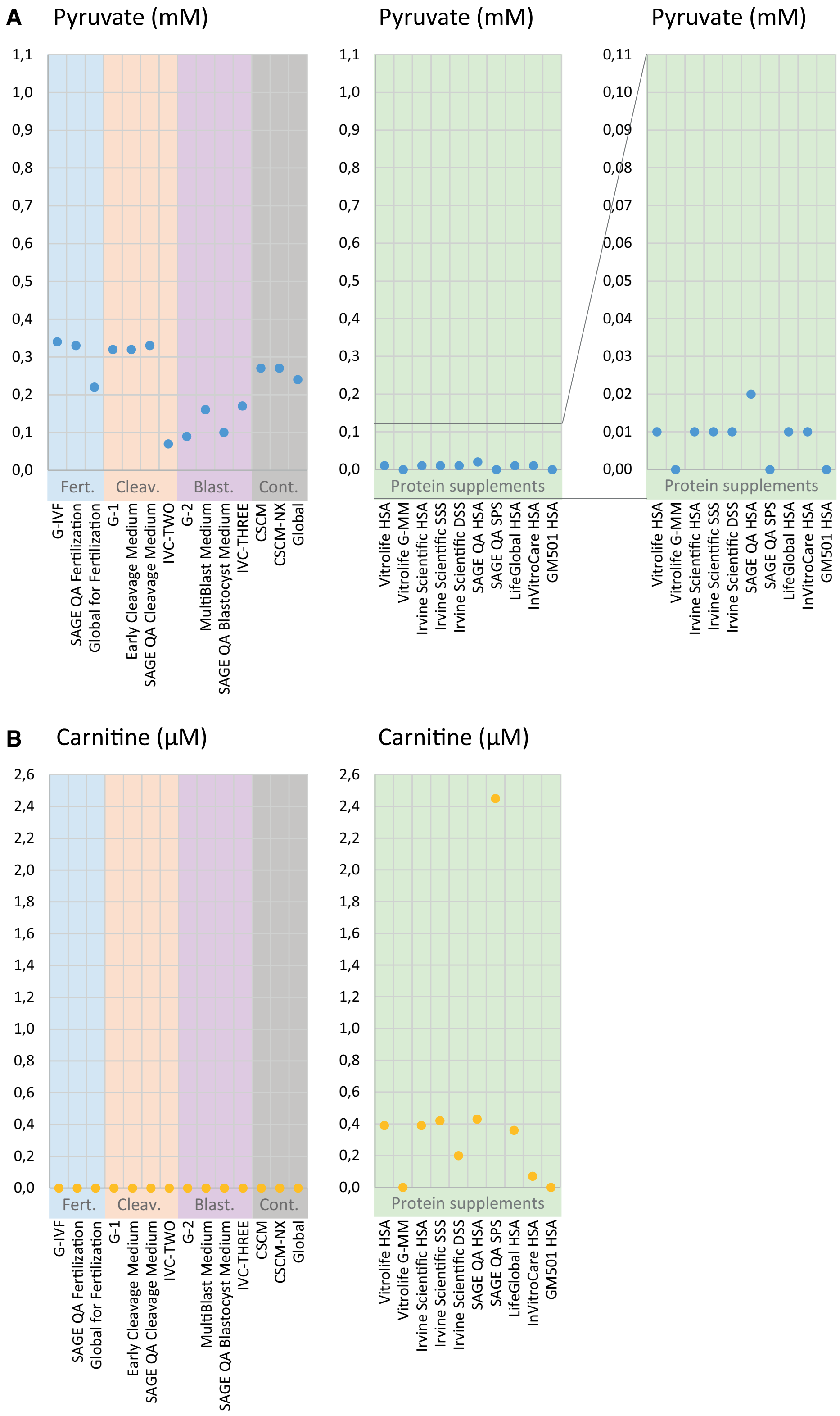

Supplementary Figure S3. Concentrations of energy sources (pyruvate and carnitine) determined in 14 unsupplemented commercial human embryo culture media and 10 protein supplements. (**A**) Pyruvate concentrations in mM. (**B**) Carnitine concentrations in µM.
