# Supplementary material for: The composition of commercially available human embryo culture media
Source: Hum Reprod. 2024 Nov 25;40(1):30–40. doi: 10.1093/humrep/deae248 (PMC11700899; doi:10.1093/humrep/deae248)
Supplement: deae248_Supplementary_Figure_S4 [file deae248_supplementary_figure_s4.pdf]

OXFORD
human reproduction

Human Reproduction, 2024, 1–1
https://doi.org/10.1093/humrep/deae248
Supplementary Data

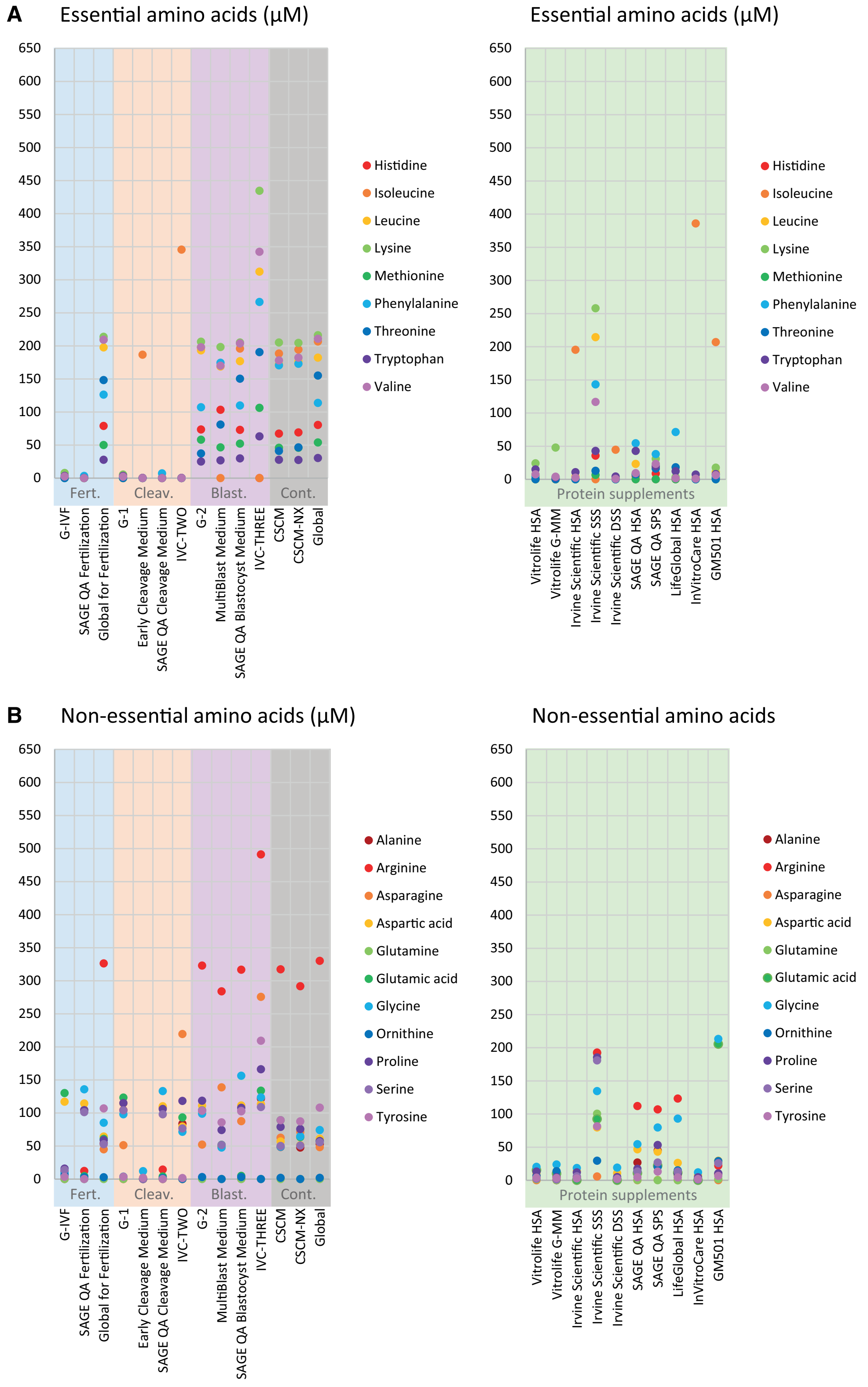

Supplementary Figure S4. Concentrations of amino acids determined in 14 unsupplemented commercial human embryo culture media and 10 protein supplements. (**A**) Essential amino acid concentrations in µM. (**B**) Non-essential amino acid concentrations in µM. Glycine concentrations in these samples were all below 214 µM.
