# Supplementary material for: The composition of commercially available human embryo culture media
Source: Hum Reprod. 2024 Nov 25;40(1):30–40. doi: 10.1093/humrep/deae248 (PMC11700899; doi:10.1093/humrep/deae248)
Supplement: deae248_Supplementary_Figure_S5 [file deae248_supplementary_figure_s5.pdf]

Human Reproduction, 2024, 1–1
https://doi.org/10.1093/humrep/deae248
Supplementary Data

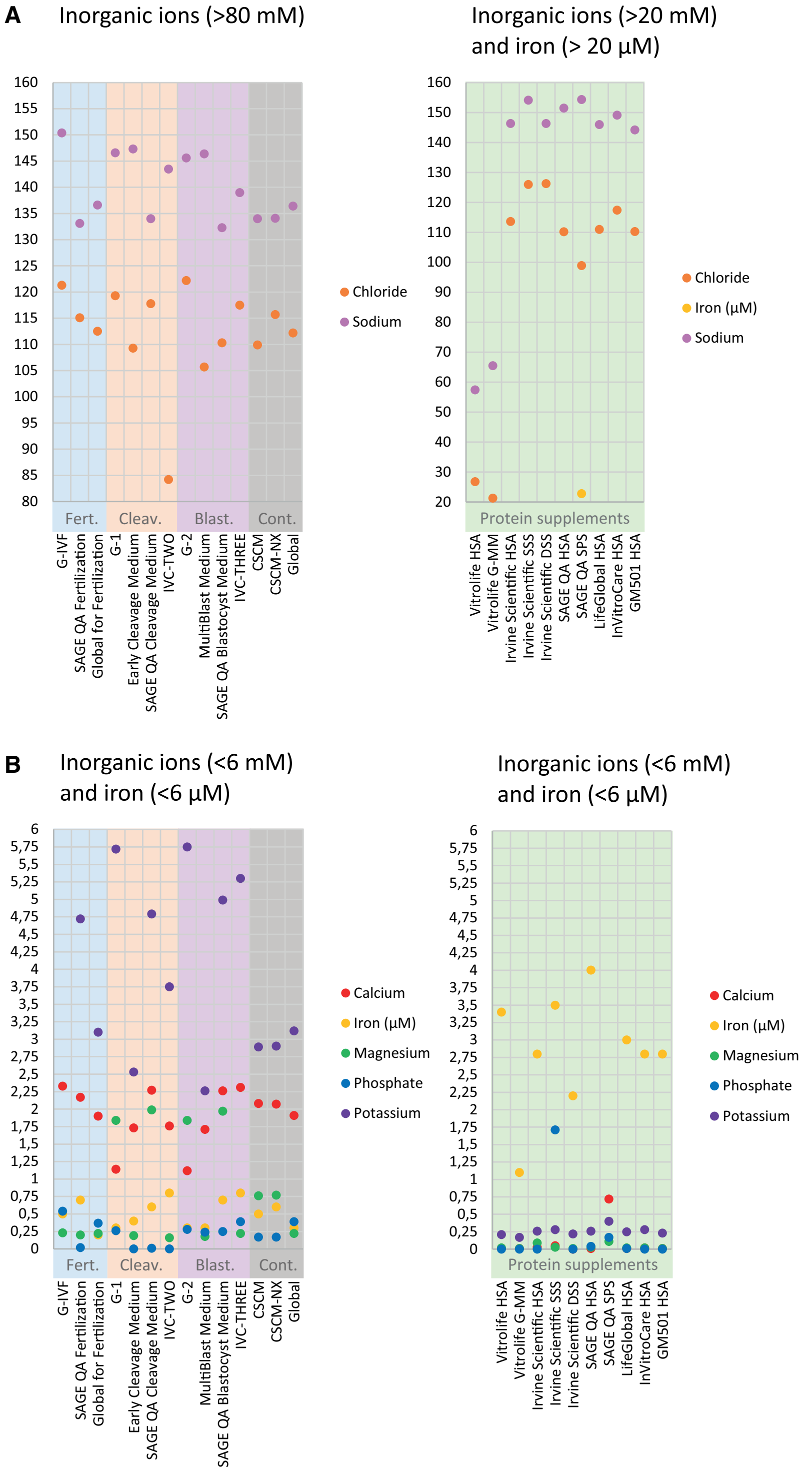

Supplementary Figure S5. Concentrations of inorganic ions (calcium, chloride, magnesium, phosphate, potassium, and sodium) and iron determined in 14 unsupplemented commercial human embryo culture media and 10 protein supplements. (**A**) Chloride and sodium concentrations in mM. (**B**) Calcium, magnesium, phosphate, potassium concentrations in mM, and iron concentrations in µM.
